# Risk Factors for Mortality in Children with Congenital Heart Disease Delivered at a Brazilian Tertiary Center

**DOI:** 10.21470/1678-9741-2018-0174

**Published:** 2018

**Authors:** Luciane Alves Rocha, Sthefane Catib Froio, Célia Camelo Silva, Simone de Araujo Negreiros Figueira, José Cícero Stocco Guilhen, Ruth Guinsburg, Edward Araujo Júnior

**Affiliations:** 1 Discipline of Fetal Medicine, Department of Obstetrics, Escola Paulista de Medicina da Universidade Federal de São Paulo (EPM-UNIFESP), São Paulo, SP, Brazil.; 2 Discipline of Cardiology, Department of Medical Clinic, Escola Paulista de Medicina da Universidade Federal de São Paulo (EPM-UNIFESP), São Paulo, SP, Brazil.; 3 Discipline of Neonatology, Department of Pediatrics, Escola Paulista de Medicina da Universidade Federal de São Paulo (EPM-UNIFESP), São Paulo, SP, Brazil.; 4 Discipline of Cardiovascular Surgery, Department of Surgery, Escola Paulista de Medicina da Universidade Federal de Sao Paulo (EPM-UNIFESP), Sao Paulo, SP, Brazil.

**Keywords:** Risk Factors, Heart Defects, Congenital, Infant, Newborn, Mortality

## Abstract

**Objective:**

This study aims to investigate the incidence of postnatal diagnosis of congenital heart disease (CHD) and the predictive factors for hospital mortality.

**Methods:**

This retrospective cohort study was conducted at a Brazilian tertiary center, and data were collected from medical records with inclusion criteria defined as any newborn with CHD diagnosed in the postnatal period delivered between 2015 and 2017. Univariate and multivariate analyses were performed to determine the potential risk factors for mortality.

**Results:**

During the 3-year period, 119 (5.3%) children of the 2215 children delivered at our institution were diagnosed with CHD. We considered birth weight (*P*=0.005), 1^st^ min Apgar score (*P*=0.001), and CHD complexity (*P*=0.013) as independent risk factors for in-hospital mortality. The most common CHD was ventricular septal defect. Indeed, 60.5% cases were considered as "complex" or "significant" CHDs. Heart surgeries were performed on 38.9% children, 15 of whom had "complex" or "significant" CHD. A mortality rate of 42% was observed in this cohort, with 28% occurring within the initial 24 h after delivery and 38% occurring in patients admitted for heart surgery.

**Conclusion:**

The postnatal incidence of CHD at our service was 5.3%. Low 1^st^ min Apgar score, low birth weight, and CHD complexity were the independent factors that affected the hospital outcome.

**Table:** 

Abbreviations, acronyms & symbols	
**CHD**	**= Congenital heart disease**
**ECMO**	**= Extracorporeal membrane oxygenation**
**EPM-UNIFESP**	**= Paulista School of Medicine, Federal University** **of São Paulo**
**RACHS**	**= Risk adjustment for congenital heart surgery**

## INTRODUCTION

Congenital heart diseases (CHDs), the leading abnormalities in fetuses, are six times more common than chromosomal abnormalities and four times more common than neural tube defects^[1]^. The incidence of CHD with intrauterine diagnosis ranges from 2.4% to 54%^[2-7]^. However, some countries witness high incidence of CHD possibly due to the implementation of an organized policy to perform ultrasound heart screening^[8-10]^. In Brazil, CHDs are the main cause of death among infants with congenital abnormality, and the implementation of health public policies targeting such population may decrease infant mortality, as occurred in developed countries^[11]^.

A comprehensive assessment of the fetal heart optimizes the diagnosis of CHD, offering appropriate prenatal and postnatal planning and facilitating an improvement in neonatal morbidity and surgical outcome^[10,12-17]^. Newborns with postnatal diagnosis could have unfavorable outcomes because symptoms and cardiovascular impairment may develop at home or in a community hospital, further increasing the morbidity and mortality rates.

Previously, a few studies focused on surgical and hospital outcomes^[9]^, encouraging us to document our experience with newborns with CHD at our service. Thus, the aim of our study was to define the current postnatal incidence of CHD at one Brazilian referral center and to determine the risk factors that may affect the hospital outcome.

## METHODS

This historical cohort study was conducted at one referral center [São Paulo Hospital at Paulista School of Medicine, Federal University of São Paulo (EPM-UNIFESP, São Paulo, Brazil)] having expertise in fetal and pediatric echocardiography and critical care for complex CHDs in Southeast Brazil, however, without extracorporeal membrane oxygenation (ECMO).

Data were collected from the medical records of pregnant women and their newborns diagnosed with CHD. The study protocol was approved by the Ethics Committee of UNIFESP.

In this study, the inclusion criteria were newborns with postnatal diagnosis of CHD delivered at our service between January 2015 and December 2017, regardless of the center where the prenatal follow-up was conducted. Patients with inadequate echocardiographic data were excluded from the final analysis. We used the classification system of fetal heart diseases based on the complexity of anatomical heart abnormalities^[18,19]^. "Complex CHDs" included heterotaxy or atrial isomerism, atresia or severe hypoplasia of a valve or chamber (hypoplastic left heart syndrome, pulmonary atresia, tricuspid atresia, aortic atresia, mitral atresia, and Ebstein's anomaly), and abnormalities of the valve inlet or outlet (complete atrioventricular septal defect, truncus arteriosus, double inlet left or right ventricle, and double outlet left or right ventricle congenitally corrected transposition of the great arteries). "Significant CHDs" included transposition of the great vessels, tetralogy of Fallot, large ventricular septal defect, coarctation of the aorta, aortopulmonary window, critical aortic or pulmonary stenosis, partial atrioventricular septal defect, total anomalous pulmonary venous connection, and tricuspid valve dysplasia (no Ebstein's anomaly). "Minor CHDs" included small ventricular septal defect and less severe aortic or pulmonary stenosis, whereas dysrhythmias, cardiomyopathies, secondary dextrocardia/levocardia, pulmonary sequestration, and patent ductus arteriosus were classified as "other CHDs"^[18,19]^.

Furthermore, newborns were categorized undergoing surgery depending on the study of risk adjustment for in-hospital mortality of children undergoing congenital heart surgery (designated as RACHS - Risk adjustment for congenital heart surgery)^[20]^. RACHS can be used in the comparative assessment of outcomes among institutions to guide quality improvement efforts. In this study, independent variables included gestational age at delivery (weeks), birth weight (g), prematurity, 1^st^ and 5^th^ min Apgar score, karyotype analysis, presence of extracardiac malformations, type of delivery, type of CHD, clinical and surgical treatments for CHD, need for mechanical ventilation, antibiotics, and vasoactive drugs, and the dependent variable was hospital mortality.

Means and standard deviations were calculated for quantitative variables, and percentage and absolute values were described for qualitative variables. Subsequently, an inferential analysis of the study variables was conducted. The unpaired Student's t-test was used for quantitative variables and chi-square or Fisher's exact test for qualitative variables. For all analyses, *P*-values of <0.05 was considered statistically significant. All baseline variables with univariate analysis exhibiting *P*<0.10 were selected for logistic regression analysis.

The analyses were performed using the program STATA/IC 12.1 (College Station, TX, USA) for MacBook (Apple Inc., Cupertino, CA, USA).

## RESULTS

During the 3-year study period, 2215 children were born at our institution, of whom 119 (5.3%) were diagnosed with CHD, and no child was excluded in this study. Table 1 summarizes the study population, stating that the prenatal diagnosis of CHD was 84.8%.

**Table 1:** Baseline characteristics of patients (n=119).

Variables	
Maternal age in years, mean ± SD	30±7.9
Maternal age ≥35 years, n (%)	36 (31.5)
Prenatal diagnosis of CHD, n (%)	101 (84.8)
Gestational age at delivery in weeks, mean ± SD	36.8±2.7
Prematurity, n (%)	38 (31.9)
Cesarean, n (%)	83 (69.7)
Birth weight in gram, mean ± SD	2512±789
Birth weight ≤ 2500 g, n (%)	56 (47)
1^st^ min Apgar score, mean ± SD	6.4±2
5^th^ min Apgar score, mean ± SD	7.8±2
Multiple fetal malformations, n (%)	65 (54)
Neurological system	26
Facial	17
Respiratory system	3
Gastrointestinal tract	9
Genital	3
Abdominal	7
Urinary tract	9
Arms and limbs	24
Chromosomal abnormalities, n (%)	32 (26.8)
Trisomy 21	12
Trisomy 18	11
Trisomy 13	7
Others	2

CHD=congenital heart disease; SD=standard deviation

The mean maternal age was 30±7.9 years, and 31.5% were aged ≥ 35 years. The mean gestational age at delivery was 36±5 (range, 34-39) weeks. Approximately 31% children were prematurely born, and 47% had low birth weight. In addition, neurological disorders (48.8%) were found to be the most common malformations associated with CHD, and trisomy 21 (37.5%) and trisomy 18 (34.3%) were the leading chromosomal abnormalities.

We considered 72 (60.5%) cases as "complex" and "significant" according to the classification system of CHDs based on the complexity of anatomical heart abnormalities. Our study reported ventricular septal defect to be the most common CHD (Table 2). In the delivery room, resuscitation was conducted in 36.1% of the children; of these, 14.3% required intubation. Most children needed mechanical ventilation and vasoactive drugs during hospitalization (59.7% and 50.3%, respectively). Furthermore, heart surgery was performed in 38.9% of the children (Table 3).

**Table 2:** Congenital heart disease by the classification system according to the complexity of anatomical heart abnormalities (n=119).

Congenital heart disease	Frequency (n)
Complex	51
Heterotaxy or atrial isomerism	2
Hypoplastic left heart syndrome	11
Pulmonary atresia	8
Tricuspid atresia	2
Ebstein’s anomaly	2
Truncus arteriosus	2
Complete atrioventricular septal defect	17
Double outlet of right ventricle	6
Ectopia cordis	1
Significant	21
Tetralogy of Fallot	6
Transposition of the great vessels	2
Critical pulmonary stenosis	1
Coarctation of the aorta	11
Total anomalous pulmonary venous connection	1
Minor	40
Small ventricular septal defect	28
Atrial septal defect	11
Less severe pulmonary stenosis	1
Others	7
Cardiomyopathies	1
Secondary dextrocardia/levocardia	3
Persistent ductus arteriosus	3

**Table 3:** Interventions performed in the study population (n=119).

Variables	N(%)
Resuscitation in the delivery room, n (%)	43 (36.1)
Positive pressure ventilation, n (%)	42 (35.3)
Intubation in the delivery room, n (%)	17 (14.3)
Mechanical ventilation, n (%)	71 (59.7)
Parenteral nutrition, n (%)	56 (47)
Cardiac surgery, n (%)	46 (38.9)
Antibiotics, n (%)	27 (23)
Vasoactive drug, n (%)	59 (50.3)

**Fig. 1:**
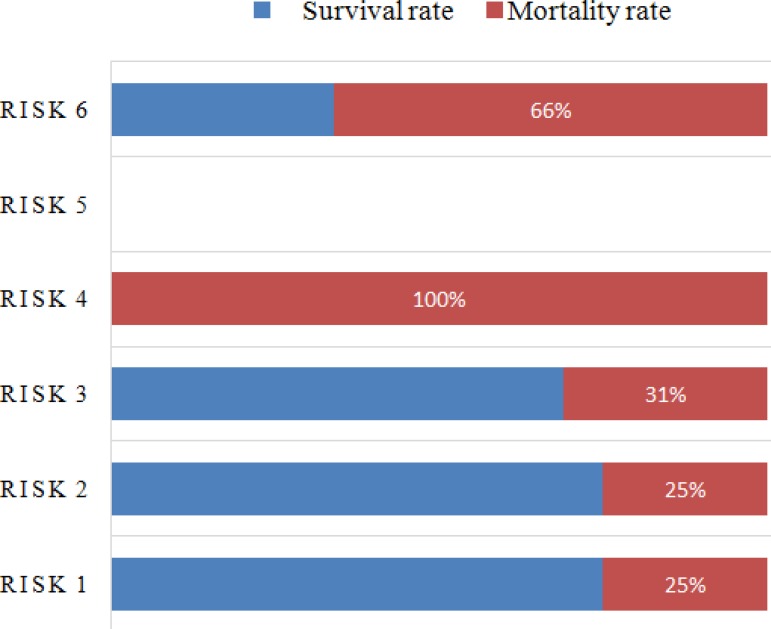
The frequency of surgical cases and mortality based on the study RACHS (n=46).

Figure 1 shows the frequency of surgical cases based on the study of risk adjustment for in-hospital mortality of children after undergoing surgery for CHD (designated RACHS). In addition, mortality can be observed for each risk category. The highest mortality rate was observed in category 6 (66.7%). Among the patients admitted for surgery but who died, a tendency of correlation with the complexity of the disease was noted (*P*=0.08).

Mortality was observed in 42% cases (n=50), of which 14 (28%) occurred within the initial 24 hours after delivery, and the average time of death was 19 days. In addition, 18 (38%) cases of death were recorded among patients admitted for heart surgery, 15 of whom had a "complex" or "significant" CHD. Because variables such as weight, prenatal diagnosis, 1^st^ min Apgar score, chromosomal abnormalities, and CHD complexity correlate with mortality (*P*<0.10) in the univariate analysis, they were selected for logistic regression. Finally, we considered low birth weight (*P*=0.005), low 1^st^ min Apgar score (*P*=0.001), and CHD complexity (*P*=0.013) as independent risk factors for hospital mortality (Table 4).

**Table 4:** Mortality rates in the study population and associated factors (n=50).

Variables	Death	*P*
Maternal age in years, mean ± SD	30.6 ± 7.1	0.49^(1)^
Gestational age at delivery in weeks, mean ± SD	36.4 ± 3.3	0.22^(1)^
Prematurity, n (%)	16 (32)	0.98^(2)^
Birth weight in gram, mean ± SD	2228.5±863	0.0007^(1)^^/^^(*)^
Prenatal diagnosis, n (%)	47 (94)	0.02^(2)^^/^^(*)^
Cesarean, n (%)	37 (74)	0.39^(2)^
1^st^ min Apgar score, mean ± SD	6.4±2	<0.0001^(1)^^/^^(*)^
Multiple fetal malformations, n (%)	29 (58)	0.34^(2)^
Chromosomal abnormalities, n (%)	18 (36)	0.08^(2)^^/^^(*)^
Complexity of the CHD, n (%)		0.08^(3)^^/^^(*)^
Complex	28 (56)	
Significant	7 (14)	
Minor	12 (24)	
Others	3 (6)	

(1) Student’s t-test.

(2) Chi-square test.

(3) Fisher’s exact test.

* Variables selected for logistic regression test.

CHD=congenital heart disease; SD=standard deviation

## DISCUSSION

This study demonstrated that the current frequency of postnatal detection of CHD at our service is 5.3%. The variables low 1^st^ min Apgar score, low birth weight, and CHD complexity were independent factors that affected hospital mortality.

Similar to previous studies, we established that the postnatal incidence of CHD is high at our center^[21,22]^ possibly due to the absence of laws that facilitate the interruption of gestation in Brazilian patients with CHD^[9,15,17]^. Moreover, patients are referred to a tertiary center after prenatal diagnosis, thereby increasing the incidence of CHD at our institute. Additionally, in the univariate analysis, the prenatal diagnosis was associated with death probably due to the same fact that our institution is tertiary. A study comparing the surgical results of between children with intrauterine diagnosis and postnatal diagnosis may clarify such finding.

The higher prevalence of "complex" and "significant" CHD can also be attributed to the fact that our hospital is a reference center for CHD. Besides, the routine use of Doppler echocardiography has increased the diagnosis of "minor" defects (*i.e*., small ventricular septal defect, milder forms of pulmonary stenosis, and atrial septal defect) in asymptomatic children. In fact, some studies have reported a high frequency of ventricular septal defect^[22]^, as established in our study.

Reportedly, low birth weight is associated with increased mortality rate in patients admitted for heart surgery^[23,24]^. Remarkably, we determined that low birth weight was an independent risk factor for hospital mortality regardless of patients being admitted for surgery or not. In addition, other studies have established a correlation between prematurity and increased mortality rate, rather than low birth weight; however, such association was not determined in our analysis^[25,26]^. Low birth weight is also often associated with other major congenital anomalies, which might affect morbidity and mortality. Consequently, such cases are the most complicated and severe ones in the delivery room, presenting an increased risk of having a lower 1^st^ min Apgar score (other independent risk factors for mortality that were found).

Previous studies have reported early mortality rates ranging from 10% to 42%^[22,27]^; however, studies that reported the lowest mortality rate included no patients with functionally univentricular physiology and all their patients could undergo surgery at a single stage^[27]^. The mortality rate of our patients admitted for heart surgery was 38%, which could be attributed to the high complexity of heart diseases at our service (85% of cases operated) with the majority of patients with functionally univentricular physiology. The mortality rate was slightly higher (66.7%) in our study than the expected mortality rate (47.7%) for RACHS risk 6^[20]^. Probably, multifactorial causes justify our suboptimal results. The absence of ECMO in our institution^[28]^, as well as not measured aspects related to hospital infra-structure, human resources or children nutritional state may be associated. Despite of this, such results showed us that we need to improve the quality of the data collection in order to identify flaws during the preoperative, intraoperative and/or postoperative course.

However, our study had several drawbacks, many of which are attributed to the retrospective study design. Moreover, we did not collect the prenatal echocardiographic data and used the hospital mortality as an outcome. The lack of representative ambulatory data precluded comprehensive analysis of the prognosis. Furthermore, the absence of intraoperative and postoperative data as well as specific causes of the mortality prevented us from differentiating the primary cause of mortality.

## CONCLUSION

In conclusion, this study reports that the postnatal incidence of CHD at our service was 5.3% and that low 1^st^ min Apgar score, low birth weight, and CHD complexity are independent factors, affecting hospital outcome. The results of this study could contribute to the development of policies that improve mortality rates.

**Table:** 

**Authors’ roles & responsibilities**	
LAR	Writing and statistical analysis; final approval of the version to be published
SCF	Collect data; final approval of the version to be published
CCS	Collect data; final approval of the version to be published
SANF	Critical review; final approval of the version to be published
JCSG	Critical review; final approval of the version to be published
RG	Supervision; final approval of the version to be published
EAJ	Supervision; final approval of the version to be published
